# Meat consumption and risk of breast cancer in the UK Women's Cohort Study

**DOI:** 10.1038/sj.bjc.6603825

**Published:** 2007-05-29

**Authors:** E F Taylor, V J Burley, D C Greenwood, J E Cade

**Correction to**: *British Journal of Cancer* (2007) **96**, 1139–1146. doi:10.1038/6603689

Owing to an author error, the numbers in the columns headed ‘person years’ in Tables 2–4 in the above paper are incorrect. The mean person years should be adjusted by adding 0.8 to each value and the standard deviations should not have been included. All other results are correct.

